# Complete sources of cluster variation on the risk of under-five malaria in Uganda: a multilevel-weighted mixed effects logistic regression model approach

**DOI:** 10.1186/s12936-023-04756-3

**Published:** 2023-10-19

**Authors:** Charles Natuhamya, Fredrick Makumbi, Aggrey David Mukose, John M. Ssenkusu

**Affiliations:** Makerere University School of Public Health, P.O Box 7062, Kampala, Uganda

**Keywords:** Cluster variation, Mixed-effects model, Multi-level weighting, Under-five malaria, Uganda

## Abstract

**Background:**

Malaria, a major cause of mortality worldwide is linked to a web of determinants ranging from individual to contextual factors. This calls for examining the magnitude of the effect of clustering within malaria data. Regrettably, researchers usually ignore cluster variation on the risk of malaria and also apply final survey weights in multilevel modelling instead of multilevel weights. This most likely produces biased estimates, misleads inference and lowers study power. The objective of this study was to determine the complete sources of cluster variation on the risk of under-five malaria and risk factors associated with under-five malaria in Uganda.

**Methods:**

This study applied a multilevel-weighted mixed effects logistic regression model to account for both individual and contextual factors.

**Results:**

Every additional year in a child’s age was positively associated with malaria infection (AOR = 1.42; 95% CI 1.33–1.52). Children whose mothers had at least a secondary school education were less likely to suffer from malaria infection (AOR = 0.53; 95% CI 0.30–0.95) as well as those who dwelled in households in the two highest wealth quintiles (AOR = 0.42; 95% CI 0.27–0.64). An increase in altitude by 1 m was negatively associated with malaria infection (AOR = 0.98; 95% CI 0.97–0.99). About 77% of the total variation in the positive testing for malaria was attributable to differences between enumeration areas (ICC = 0.77; p < 0.001).

**Conclusions:**

Interventions towards reducing the burden of under-five malaria should be prioritized to improve individual-level characteristics compared to household-level features. Enumeration area (EA) specific interventions may be more effective compared to household specific interventions.

## Background

Malaria, a major cause of mortality worldwide [1] is caused by protozoa of the genus Plasmodium [2]. Within the two years of the start of COVID-19 pandemic, malaria endemic countries, Uganda inclusive, reported more than 101 million cases [3]. Uganda emerges as one of the six countries that account for more than half of all malaria cases worldwide [4] where children under five years of age are the most vulnerable group [5].

Malaria is linked to a web of determinants ranging from individual to contextual [6–9]. Hence, it’s vital to take advantage of the opportunity provided by malaria data, to critically examine the magnitude of the effect of clustering of data points [10].

Issues that researchers usually overlook while modelling multilevel data based on complex survey design are; use of final survey weights (single level weights that are only appropriate for single level analysis) instead of multilevel weights or level-specific weights [11] and ignoring cluster variation on the risk of study outcomes/diseases [12]. This most likely produces biased estimates, misleads inference and lowers study power. Besides, it is critical to consider several metrics like ICC as it was the case of this study, to identify risk factors of disease and derive appropriate public health interventions while analysing data from multilevel study designs [13]. Failure to do so can lead to inappropriate interventions for prevention and control of diseases like malaria and associated adverse consequences. Although some studies based on a multilevel design have adjusted for clustering in the data, a number of them have not considered determining the complete sources of cluster variation on malaria which may be an important statistic to guide critical levels of public health interventions and future study designs [14, 15].

The objective of this study was, therefore, to determine the complete source of cluster variation on the risk of malaria, and to identify risk factors associated with under-five malaria in Uganda.

## Methods

### Data source and study population

**Fig. 1:**
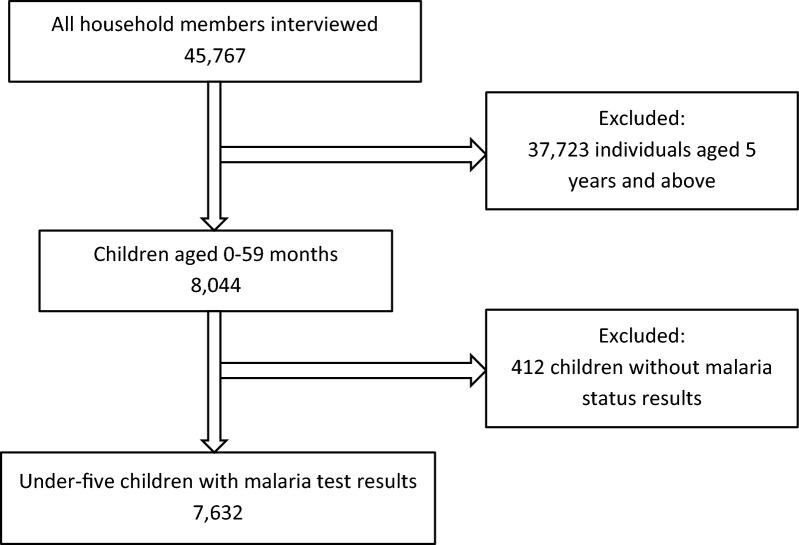
Flow chart showing selection of the study participants

This study made use of secondary data based on a two-stage cluster and stratified sampling technique from the Uganda Malaria Indicator Survey (UMIS) of 2018/19. The first stage of sampling involved selecting sample points (clusters) from the sampling frames. A total of 320 clusters were selected with probability proportional to size from the enumeration areas (EAs) covered in the 2014 National Population and Housing Census (NPHC). The second stage of sampling involved systematic selection of households. Twenty-eight households were selected from each EA, for a total sample size of 8,878 households. The primary objective of the 2018–19 UMIS is to provide up-to-date estimates of basic demographic and health indicators related to malaria. Specifically, the 2018/19 UMIS collected information on vector control interventions such as mosquito nets and indoor residual spraying of insecticides, on intermittent preventive treatment of malaria in pregnant women, on care-seeking and treatment of fever in children, and malaria knowledge, behaviour, and practices. All women age 15–49 who were either permanent residents of the selected households or visitors who stayed in the household the night before the survey were eligible to be interviewed. After a parent’s or guardian’s consent was obtained, children age 0–59 months were tested for anaemia and malaria infection. The study population consisted of 7,632 children less than 5 years of age who were tested for anaemia and malaria infection by a team of two health technicians, respectively [16]. The selection of the final study sample is as shown in Fig. 1.

### Analysis model

The dataset was first explored for preparation purposes. Before any analysis was conducted, the data were sorted, some variables recoded while other variables and some observations that were not of interest to the research problem were eliminated. Categorical variables were represented as counts and percentages. Collinearity was assessed among independent variables using a correlation matrix. Variables with correlation coefficient of 0.4 and above were not included in the same model. The survey design estimation command (svy) in Stata 15.0 (StataCorp, College Station, TX) was used to conduct descriptive analysis, accounting for the level weights. The level of statistical significance was p < 0.05 for all analyses. Overall, four multivariable models were considered; the first model neither adjusted for weighting nor cluster variation in the risk of under-five malaria; the second model only adjusted for cluster variation; the third model only adjusted for weighting; and the forth model adjusted for both weighting and cluster variation. A model was, therefore, considered to best fit the data if it had lower design factor (deft) values in general. Lower deft values are associated with lower loss of precision of model estimates [17]. The design factor (deft) was calculated as follows:

$$deft=\sqrt{deff}=\sqrt{1+rho(n-1)}$$

where; $deff$ is the design effect. $rho$ is the intra-class correlation for the variable in question. $n$ is the size of the cluster.

To assess the association between malaria infection in under-five children and individual, household, and enumeration area factors, a multilevel-weighted mixed effects logistic regression model (chosen among the four compared models as the best model) was specified to account for contextual within-household and within-EA correlations [18–20]. The model is represented as below:

$$ln\left(\frac{{p}_{ijk}}{1-{p}_{ijk}}\right)={\beta }_{0}+ {\beta }_{1}{X}_{ijk}+ {\eta }_{k}+ {\xi }_{jk}$$

where; $ln$ is the natural logarithm. ${p}_{ijk}$ is the probability of testing positive for malaria for the *i*th under 5-year-old child in household $j$ and EA $k$.

${\beta }_{0}$ is the mean log-odds of malaria across household and EA.

${X}_{ijk}$ is a level 1 covariate for the *i*th child in household $j$ and EA $k$.

${\beta }_{1}$ represents the slope associated with ${X}_{ijk}$ which represents the relationship between the level 1 covariates and the log-odds of malaria.

${\eta }_{k}$ is the random effect for EA $k$.

${\xi }_{jk}$ is the household random effect.

Bivariate multilevel weighted-mixed effects logistic regression was conducted, using each of the individual, household, and community level risk factors as predictors and malaria test result as the outcome. Individual predictors with p < 0.20 were considered for inclusion in the multivariable multilevel logistic regression models. The multivariable analysis was conducted in a sequential process resulting into several models. Model 0 (the null model) was fitted to decompose the total variance of malaria risk between the cluster and level-1 covariates. It only included the random intercept to assess EA and household contribution to malaria risk before adding explanatory variables. The null model established the degree of variance at the cluster level in order to validate the use of multilevel modeling. Model 1 contains individual (level-1) variables; model 2 has household (level-2) variables in addition to variables in model 1; model 3 includes EA (level-3) variables in addition to variables in model 2. Model 3 was selected as the final model that was used to identify factors associated with malaria risk in under-five children since it was the most complete among the three models. To measure the extent to which individuals within the same group are more similar to each other than they are to individuals in different groups, intra-class correlation coefficient (ICC) was used [21]. A higher proportion of the ICC was linked to a higher general contextual effect [22]. The formula for the ICC is presented as below:

$$ICC\; = \;\frac{V_A }{{V_A \; + \;{{\pi^2 } / 3}}}$$

where ${V}_{A}$ is the cluster or area level variance and $\pi^2 /3$ is a scalar that corresponds to the individual level variance. When the contribution to the overall ICC of a level(s) was very low (< 10%) its effect was considered insignificant and hence, the random effects component(s) at the specific level(s) was considered insignificant.

### Weighting

Since the sample for this study is a two-stage stratified cluster sample, level weights were calculated separately, based on sampling probabilities for each sampling stage and cluster. In this study, level weights were estimated using a framework for approximating level weights in Malaria Indicator Surveys (MIS) proposed by the Demographic Health Survey program [11]. The framework required data that is included in the publicly available UMIS datasets and final report.

## Results

### Characteristics of children aged 0–59 months

Overall, children were evenly distributed by sex. On average, children were aged 29.2 (SD = 17.3) months. Slightly more children were anaemic 3939 (51.6%). A higher proportion of households were headed by males 5478 (71.8%) and in the two lowest wealth quintiles 4,385 (57.5%) with at least five members 5880 (77.1%). Most households did not have electricity 4921 (64.5%), had at least one bed net 6666 (87.4%), but had not sprayed their dwellings within the last 12 months of the survey 6601 (86.5%) and were in rural areas 5516 (72.3%). Finally, most mothers had attained at least primary level of education 5002 (78.7%). The rest of the results are presented in Table 1.

**Table 1:** Distribution of under-five children by selected background characteristics

Background characteristics	Category	Count	Percent
Sex of child	Male	3,870	50.7
	Female	3,762	49.3
Age of child	< 1	1,502	19.7
	1	1,441	18.9
	2	1,502	19.7
	3	1,608	21.1
	4	1,579	20.7
Anaemia status	Not anaemic	3,691	48.4
	Anaemic	3,939	51.6
Sex of HH head	Male	5,478	71.8
	Female	2,152	28.2
Wealth index	Poor	4,385	57.5
	Middle	1,227	16.1
	Rich	2,018	26.5
Has electricity	No	4,921	64.5
	Yes	2,709	35.5
Has bed net	No	964	12.6
	Yes	6,666	87.4
HH size	Below 5	1,750	22.9
	5 & above	5,880	77.1
Residence	Urban	1,453	19.0
	Rural	5,516	72.3
	Refugee	661	8.7
HH sprayed	No	6,601	86.5
	Yes	989	13.0
	Don’t know	40	0.5
Mother’s education	No education	1,354	21.3
	Primary	3,647	57.4
	Secondary & above	1,355	21.3

### Regional variation in prevalence of malaria among the under-five children

**Fig. 2:**
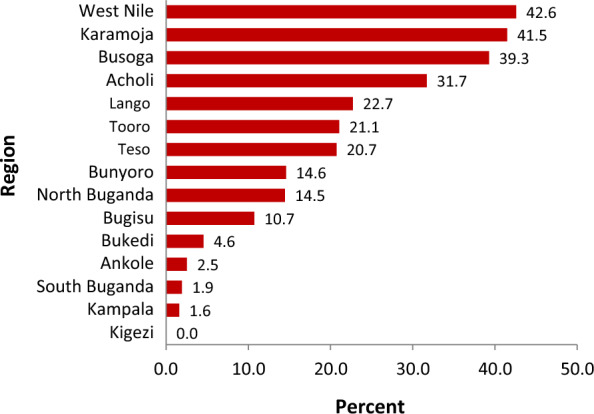
Weighted regional ranking of prevalence of under-five malaria

Malaria prevalence showed great variability across regions. Of the 15 statistical regions in Uganda, five of them had malaria prevalence above the national average of 21.1%. Prevalence was highest in the West Nile region (42.6%), followed by Karamoja region (41.5%), Busoga region (39.3%), Acholi region (31.7%) and Lango region (22.7%) as shown in Fig. 2.

### Factors associated with malaria risk in under-five children

Table 2 shows results of the multivariable multilevel-weighted mixed effects logistic regression modelling at various levels. It is by this model that risk factors of under-five malaria were determined. At the individual level, every additional year in a child’s age was associated with 42% higher odds of malaria infection (AOR = 1.42; 95% CI 1.33–1.52). Also, children whose mothers had at least a secondary school education had about 47% lower odds of malaria infection compared to those whose mothers were uneducated (AOR = 0.53; 95% CI 0.30–0.95).

**Table 2:** Factors associated with prevalence and risk of malaria in under-five children

Variable	Model 1 (Individual level)			Model 2 (HH level)			Model 3 (EA level)		
	AOR	(95% CI)		AOR	(95% CI)		AOR	(95% CI)	
Sex of child									
Female	1.02	0.83	1.24	1.00	0.82	1.22	0.98	0.80	1.21
Age of child	1.40**	1.31	1.50	1.41**	1.32	1.51	1.42**	1.33	1.52
Mother's education									
Primary	0.93	0.67	1.28	0.94	0.67	1.30	0.94	0.66	1.33
Secondary and above	0.50*	0.29	0.86	0.59*	0.33	1.02	0.53*	0.30	0.95
Has bed net									
Yes	0.81	0.60	1.09	0.82	0.60	1.11	0.77	0.56	1.06
Wealth Index									
Middle	–	–	–	1.00	0.68	1.46	1.03	0.70	1.52
Rich	–	–	–	0.41**	0.27	0.62	0.42**	0.27	0.64
HH sprayed									
Yes	–	–	–	1.07	0.55	2.09	1.05	0.53	2.08
Don’t know	–	–	–	1.38	0.40	4.70	1.37	0.42	4.42
Cluster altitude	–	–	–	–	–	–	0.98**	0.97	0.99

AOR: Adjusted odds ratio, CI: Confidence interval, EA: Enumeration area, HH: Household

^**^p < 0.001, *p < *0.05*

At household level, children who dwelled in households in the two highest wealth quintiles had lower odds of malaria infection compared to those in the two lowest wealth quintiles (AOR = 0.42; 95% CI 0.27–0.64). At enumeration area level, an increase in altitude by 1 m was associated with slightly lower odds of under-five malaria infection (AOR = 0.98; 95% CI 0.97–0.99).

The ‘sex of child’ variable was maintained in the final model regardless of its statistical insignificance because of its biological importance. Variables ‘has bed net’ and ‘HH sprayed’ were maintained because of their relational importance to the research problem, according to previous research literature.

### Measure of sources of cluster variation in the risk of malaria

**Fig. 3:**
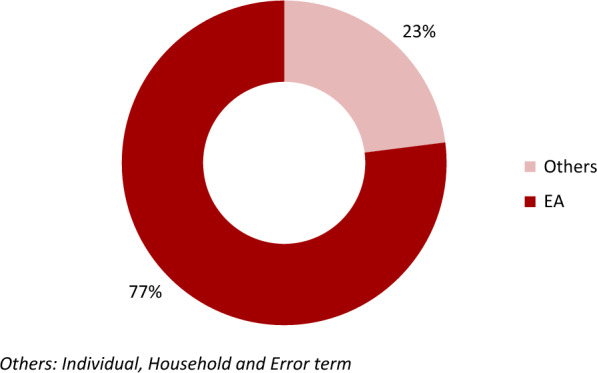
Proportional contribution of ICC by the hierarchical levels of the data

Figure 3 shows the proportional contribution of ICC across levels of the nested data and the error term. This informed the choice of the random effect term that was included in the mixed effects models. That is, EA.

The EA random effect estimate for measuring variation in Table 3 shows significant variation in under-five risk of malaria. The random effect of Model-0 (null model) shows that there was statistically significant variation in the odds of a positive test for malaria across EAs (variance = 11.16; 95% CI 8.55–14.56). The ICC value for a positive malaria test between EAs (ICC = 0.77; p < 0.001) indicates that 77% of the total variance in the positive testing for malaria was attributable to differences between EAs. Hence variation in the risk of under-five malaria was mainly attributed to EAs compared to households. In addition, moving from Model-0 to Model-3, area variance reduced by 29.6% (from 11.16 to 7.86). Area variance steadily reduced as more fixed effects were added into the model. The percentage reduction in area variance in each model equates the percent of variance contributed by fixed effects at the specific level.

**Table 3:** Measures of EA level variations in the risk of under-five malaria

Random effects	Model-0	Model-1	Model-2	Model-3
	Estimate (95% CI)	Estimate (95% CI)	Estimate (95% CI)	Estimate (95% CI)
Area variance	11.16**	11.56**	10.66**	7.86**
	(8.55–14.56)	(8.74–15.27)	(8.02–14.17)	(5.82–10.61)
ICC	0.77**	0.44**	0.29**	0.18**

CI: Confidence interval, ICC Intra-class correlation coefficient

^**^p < *0.001*

Moreover, the variation across EAs remained statistically significant throughout the three models (1, 2, and 3). The ICC values showed heterogeneity between EAs. The ICC values in models 1, 2 and 3 indicate that; 44% was attributable to EAs differences after adjusting for individual factors, 29% after adjusting for household factors and 18% after adjusting for EA factors as indicated in Table 3.

## Discussion

Identifying risk factors of malaria is critical in designing interventions and evaluating existing ones. This study did not find any sex difference among children under-five years of age with malaria. These results are consistent with similar studies based on multilevel analysis in Ethiopia [23] and Nigeria [24]. In addition, according to this study’s findings, children with educated mothers were less likely to have malaria compared to those whose mothers were uneducated. These results are similar to previous findings where prevalence of under-five malaria drastically decreases with increase in a mother’s education [25] since education has previously been associated with use of malaria prevention measures like mosquito nets [16] as educated mothers are more prone to using these preventive measures.

At the household level, wealth index was significantly associated with malaria parasitemia. These finding are in agreement with previous studies [25, 26]. This is partly due to the fact that a household’s economic status that can be represented by wealth index affects other factors like housing conditions which in turn have an effect on malaria prevalence in the affected households [16]. Also, the nature of residence like in this study, has been found to be significantly associated with under-five malaria infection like it is the case with other studies [26, 27].

Determining sources of cluster variation is important in designing future study designs and identifying effective and efficient levels at which interventions should be designed. The study presented a significantly higher ICC at EA level compared to other levels. Computing the ICC to estimate power and sample sizes is problematic given the difficulty in estimating variances a priori. Therefore, the ICC is typically estimated using the values reported in previous research [28]. Hence the calculated ICC and deft from this study can be used in computation of power and effective sample size in future studies of MIS and other similar surveys based on complex designs like Demographic and Health Survey (DHS), Multiple Indicator Cluster Survey (MICS) and Performance Monitoring for Action (PMA) surveys.

Ideally, more units should be samples at EA level compared to household level for future designs of studies based of multi-stage (specifically two-stage) sampling since ICC at EA level was higher than that at household level. These results are consistent with a study [28] that found out that as a general rule of thumb, increasing the sample size at the highest level that is, sampling more groups will do more to increase power than increasing the number of individuals in the groups. However, the problem is that increasing sample size at higher levels is more difficult and costly than increasing the sample within each group. Thus, the increases in study power may come at a substantial cost.

ICC is used in equations along with the cluster size and the number of clusters to calculate the effective sample size (ESS) which is the sample size in clustered samples as compared with the number of subjects actually enrolled in the study. As an example, proper accounting for correlation among subjects in a cluster almost always results in a net loss of power, requiring increased total subject recruitment. Increasing the number of clusters enhances power more efficiently than does increasing the number of subjects within a cluster [29].

In addition, ICC guides level-based interventions. The higher this proportion, the higher is the general contextual effect [22]. As an example, when the ICC value is lower at a specific level, then interventions need not to be done at that level but at a level where ICC is higher as it is the case of the study findings. High values of ICC therefore inform intervention to be done at specific levels [30]. This study similarly suggested interventions based at a level with higher ICC value. Instead of household-specific interventions, this study recommends EA-specific interventions.

### Study strength and limitations

The multilevel design of the MIS that allows contextual analysis was the strength of this study. The limitations were parasitaemia was diagnosed using rapid diagnostic test (RDT) which is not the gold standard. However, both microscopy (gold standard) and RDT have always yielded very close results. Also, given that the data resulted from a cross-sectional design, causal relationship between explanatory variables and under-five parasitaemia could not be guaranteed. This was a drawback concerning the prediction capability of the final model. Also, although some variables deemed important in analysis were not among the collected data, the variables available in the data were sufficient to address the study objective. Another limitation was potential recall bias because household and children characteristics were purely based on self-report by survey respondents. The potential bias was however minimal since response was only required about events from the most recent past and by eligible respondents.

## Conclusions

Individual or child characteristics were more significantly associated to malaria risk compared to household and EA characteristics. Even after accounting for individual, household and EA level fixed effects; variation remained significant at EA level hence, cluster variation was substantial at EA level. Interventions towards reducing the burden of under-five malaria should be prioritized to improve individual-level characteristics compared to household-level features. This is because more variables were significant at child-level compared to household-level. Also, EA-specific interventions towards malaria control may be more effective compared to household level interventions.

## Data Availability

The datasets analysed during the current study are publicly available in the Demographic Health Survey repository, https://dhsprogram.com/data/dataset/Uganda_MIS_2018.cfm.

## Abbreviations

AAS: Academy of Sciences Alliance
AESA: Accelerating Excellence in Science in Africa
AOR: Adjusted odds ratio
CI: Confidence interval
COVID-19: Coronavirus disease of 2019
deff: Design effect
deft: Design factor
DELTAS: Developing Excellence in Leadership, Training and Science
DHS: Demographic and Health Survey
EA: Enumeration area
ESS: Effective sample size
HH: Household
ICC: Intraclass Correlation Coefficient
ICF: Inner City Fund
MICS: Multiple Indicator Cluster Survey
MIS: Malaria Indicator Surveys
NEPAD: New Partnership for Africa’s Development Planning and Coordinating
NMCD: National Malaria Control Division
NPHC: National Population and Housing Census
PMA: Performance Monitoring for Action
RDT: Rapid diagnostic test
SD: Standard deviation
SSACAB: Sub-Saharan African Consortium for Advanced Biostatistics
TX: Texas
UBOS: Uganda Bureau of Statistics
UK: United Kingdom
UMIS: Uganda Malaria Indicator Survey
WHO: World Health Organization
